# A kick in the right direction - reduced fetal movements and stillbirth prevention

**DOI:** 10.1186/1471-2393-15-S1-A7

**Published:** 2015-04-15

**Authors:** Alexander Heazell

**Affiliations:** 1 Maternal and Fetal Health Research Centre, Manchester Academic Health Science Centre, University of Manchester, UK

## 

Multiple studies using various approaches have associated reduced fetal movements (RFM) with stillbirth and small for gestational age infants [1,2]. More recently, RFM has been linked to neurodevelopmental delay and a lack of response to treatment for hypoxic-ischaemic encephalopathy [3,4]. The relationship between adverse pregnancy or infant outcome is thought to be mediated by placental dysfunction [5]; thus RFM represents a symptom of placental insufficiency, when a placenta cannot meet the metabolic demands of the growing fetus. This hypothesis is now supported by evidence of changes in placental structure, inflammation and function in women who present with RFM [6,7]. Critically, some of these changes in placental size, structure and pathology differentiate between pregnancies with RFM that end in healthy pregnancy or adverse outcomes [8,9]. Thus, assessment of fetal growth and placental function in mothers presenting with RFM may offer new avenues to identify babies at risk of stillbirth to target intervention.

A prospective control study found that 67 out of 303 women (22%) presenting with RFM to St Mary’s Hospital, Manchester, UK had adverse perinatal outcome. Significant predictors of adverse outcome were diastolic blood pressure, estimated weight centile, liquor volume and log[human placental lactogen (hPL)] [10]. Four cases had abnormalities on cardiotocography and 20 cases had abnormal ultrasound findings and 24 had an hPL <0.8 MoM; further work is needed to predict remaining adverse pregnancy outcomes in women with RFM. Recent interest in placental biomarkers, particularly placental growth factor (PlGF), shows encouraging data to predict adverse outcomes (e.g. true fetal growth restriction) in late pregnancy and should be explored in high-risk mothers with RFM [11]. A feasibility study of 120 women found that an intensive approach to the management of RFM using a biomarker was well adhered to and women’s anxiety decreased after investigation irrespective of the management strategy [12]. Encouragingly, the rate of composite adverse perinatal outcome reduced from 29% to 12%. This indicates that a larger definitive trial should be conducted [12].

Current data, based on a large quality improvement study, suggest that mothers should be educated about reporting changes in fetal movements and units should provide standardised care including cardiotocography/non-stress test and ultrasound scan [13]. However, implementing these changes into clinical practice has been more challenging. The UK Royal College of Obstetricians and Gynaecologists (RCOG) introduced a guideline for the management of RFM in 2011 [14]. A cross-sectional survey of UK maternity units in 2013 found that 12% of units had no guideline, and where guidelines were in place they contained a median of 7/12 recommendations ranging from 3-11. Two key challenges i) to improve maternal education about fetal movements and ii) to standardise high-quality care when women present with RFM need to be addressed to reduce stillbirths using a RFM-based approach. The AFFIRM study, a stepped-wedge customised trial will address the hypothesis that education for mothers and professionals, in combination with a standard management plan can reduce stillbirth [15]. It is hoped that this strategy understanding the associations with RFM, developing effective investigations in combination with intervention will reduce stillbirth.
